# The OREGANO knowledge graph for computational drug repurposing

**DOI:** 10.1038/s41597-023-02757-0

**Published:** 2023-12-06

**Authors:** Marina Boudin, Gayo Diallo, Martin Drancé, Fleur Mougin

**Affiliations:** AHeaD team, Bordeaux Population Health Inserm U1219, Univ. Bordeaux, F-33000 Bordeaux, France

**Keywords:** Data integration, Computational models

## Abstract

Drug repositioning is a faster and more affordable solution than traditional drug discovery approaches. From this perspective, computational drug repositioning using knowledge graphs is a very promising direction. Knowledge graphs constructed from drug data and information can be used to generate hypotheses (molecule/drug - target links) through link prediction using machine learning algorithms. However, it remains rare to have a holistically constructed knowledge graph using the broadest possible features and drug characteristics, which is freely available to the community. The OREGANO knowledge graph aims at filling this gap. The purpose of this paper is to present the OREGANO knowledge graph, which includes natural compounds related data. The graph was developed from scratch by retrieving data directly from the knowledge sources to be integrated. We therefore designed the expected graph model and proposed a method for merging nodes between the different knowledge sources, and finally, the data were cleaned. The knowledge graph, as well as the source codes for the ETL process, are openly available on the GitHub of the OREGANO project (https://gitub.u-bordeaux.fr/erias/oregano).

## Background & Summary

The rapid discovery of new drugs is a particularly topical issue. The Covid-19 health crisis that we have experienced has confirmed that. However, while current standard methods are time-consuming and very expensive, drug approvals are complicated to obtain. Before this approval, the therapeutic value of drugs must be proven in clinical trials that are organised in three phases^1^. Phase I focuses on toxicological tests on the molecule, while phase II is about the evaluation of the minimum dose to be administered to obtain an effective effect, as well as the listing of the various secondary effects. Phase III is designed to measure the effectiveness of the drug; the drug is authorised or not to be produced and sold at the end of this phase. This is a complex process, especially as almost 60% of new drugs tested in clinical trials do not pass Phase II^2^. This failure rate highlights drugs that are not effective enough and/or have too many side effects compared to their benefits. The time spent on clinical trials is therefore extremely time-consuming, taking on average 10 to 15 years from Phase I to Phase III. The development of new drugs is also very costly, so the pharmaceutical industry focuses its research on diseases that will make the heavy investments profitable. This need for cost-effectiveness is an obstacle to the discovery of certain treatments, especially for rare diseases.

Thus, alternatives to this costly traditional development process have emerged. This is the case of drug repurposing (aka drug repositioning)^3^, which aims to find a new use for existing drugs or compounds. The idea is to reuse drugs that have already been approved, as well as those that have not passed the final stages of clinical trials and have a better chance to do so. This possibility opens up prospects for the identification of drugs for rare diseases in particular^4^, as drugs that have already passed the first stages of clinical trials are less expensive to bring to market. Repositioning methods are categorised into either biological or computational methods^5^. Biological methods involve discovering new information about drugs and targets and also testing, in large studies, the possibility of binding them, mostly to proteins. However, computational methods predominate in this field, and these are diverse and varied.

Computational repositioning methods exploit knowledge about individual drugs to find possible new therapeutic targets^6^. The methods used differ according to the features describing the drugs that are taken into account. Indeed, knowledge about drugs is diverse, as compounds can be described by their chemical formula, their side effects, the targets they are aimed at, the diseases they treat and many other features. Numerous resources describe portions of this body of knowledge, many of them available online^7^. However, it is difficult to easily connect knowledge from these sources, primarily because they are not formatted in the same way and do not necessarily have cross-references allowing them to be interlinked with other knowledge sources. Similarly, the knowledge sources may not be produced for the same purpose and may not cover the same subject. Nevertheless, some projects aim to overcome these issues through different data fusion methods. The principle consists of linking the data and knowledge available in the various sources into a vast knowledge resource that is made available to all through a single endpoint. For example, the *Linked Open Data cloud* initiative brings together open source knowledge bases, linking them via nodes and links. In the area of drug repositioning, a key issue is the availability of these data and knowledge because drug information is not always made available by the companies working on the drugs.

It is possible to learn new knowledge and observations about drugs from those already available. Indeed, many studies implement learning algorithms that can, from the available data, determine whether a drug is potentially capable of binding to a new target^8^. The data used by learning algorithms must be represented in such a way that machines can learn a general model. One of our assumptions is that the more information there is to characterise a drug, the better the pattern. It is therefore necessary to integrate as much drug data as possible. One of the concerns that emerges in this context is that the data are very heterogeneous and it is difficult to characterise drugs in the same way. Indeed, drugs may have one or more targets, one or more side effects, and the targets are very diverse. In order to learn effectively on such heterogeneous data, it is necessary to find a way to represent the drug data and to leverage this representation. The type of representation that can be used at this stage is a **knowledge graph**, which has been defined by Hogan *et al*. as: “*a graph of data intended to accumulate and convey knowledge of real world, whose nodes represent nodes of interest and whose edges represent links between nodes*”^9^. A knowledge graph therefore accumulates knowledge of the real world in which the nodes represent notions of interest and whose edges represent links between them. A knowledge graph is thus a set of nodes (or entities) *N* and labelled links (or relations or predicates) *L* represented as triples of the form: (*N*_*x*_, *L*_1_, *N*_*y*_). The edges describe the binary links between two nodes and they are generally oriented and meaningful. In this case, the nodes are differentiated: the subject is the source node of the relationship, the object is the target node resulting in a triplet expressed as (subject, predicate, object).

From the perspective of defining a graph for drug repositioning, it is then possible to represent each drug, target, disease or other related entities by a node in the graph and then link these nodes together.

Biomedical data are well suited to be stored in knowledge graphs because they are scattered over many knowledge sources without being linked to each other. In this context, the “*Semantic Web initiative*” offers an idealised vision of the Web, with the idea that resources on the Web should be connected by semantic links (as opposed to hyperlinks) and that the meaning of these resources should be exploitable by machines^10^. Following this paradigm, various initiatives aiming to interconnect existing knowledge sources have emerge^11,12^.

The use of knowledge graphs for drug repurposing has been shown to be effective in recent years^13–15^. The identification of new drug-target relationships is the main goal of knowledge graph-based techniques for repositioning. The different methods focus on various aspects of the knowledge graph^16,17^. Some networks are not only composed of drugs and targets. Networks comprising other types of nodes can indeed participate in the discovery of new information for drug repositioning^18–22^. Most drug targets being proteins, protein-protein interaction networks are likely to provide very relevant information, as some proteins act indirectly on each other. Drug-target pairs can also have important consequences on other proteins, thus giving useful indications for possible repositioning^23,24^. Other studies investigated on predicting drug-disease links^25^. These studies were useful for identifying new avenues of research^26^. Graphs associating drugs and their side effects greatly assisted in the prediction of drug-target relationships^27^. These different pieces of information enrich the network and maximise the chances of finding drug-target pairs that are likely to lead to drug repurposing. The next logical step was to try to interconnect all this information into a single knowledge graph^28–31^.

The OREGANO project falls within this approach. Its objective is to develop a holistic knowledge graph on drugs and related concepts for humans in order to identify possible repositionable molecules using machine learning (or more specifically deep learning) algorithms. In the context of drug repositioning, these algorithms aim to predict the probability that a link exists between two nodes in the knowledge graph based on existing links. Such algorithms have been applied in a preliminary work on a first version of the OREGANO knowledge graph^32^. The first results were promising, and the present work aims at extending and optimizing these results by means of an updated graph composed of well-integrated sources in a first step, then by optimizing the learning methods in a second step. The OREGANO project aims to fill the missing gap in terms of computational-based drug repositioning, and performs link prediction on a large knowledge graph of heterogeneous data for discovering missing molecule-target links.

Unlike similar studies, the OREGANO knowledge graph emphasizes the integration of natural compounds (i.e. herbal and plant remedies). Indeed, for three decades (1981–2010), more than 60% of drugs were developed from natural products, derivatives or natural product-like compounds^33^. Some prominent herbal prescriptions have been transformed into new drugs^34^ (e.g. Layla Tab in Korea^35^). Therefore, we hypothesize that there is a strong possibility to seek new indications for existing herbal compounds, which could lead to the development of new drugs through repurposing strategies.

To the best of our knowledge, there is no previous work on knowledge graphs incorporating together disease and drug information and natural compounds specifically used for drug repositioning. Only two studies do refer to natural compounds and graph based representations, but for other purposes^36,37^. Indeed, these studies predict links between herbs and diseases or between herbs and targets, but on networks that are not knowledge graphs.

## Methods

**Fig. 1:**
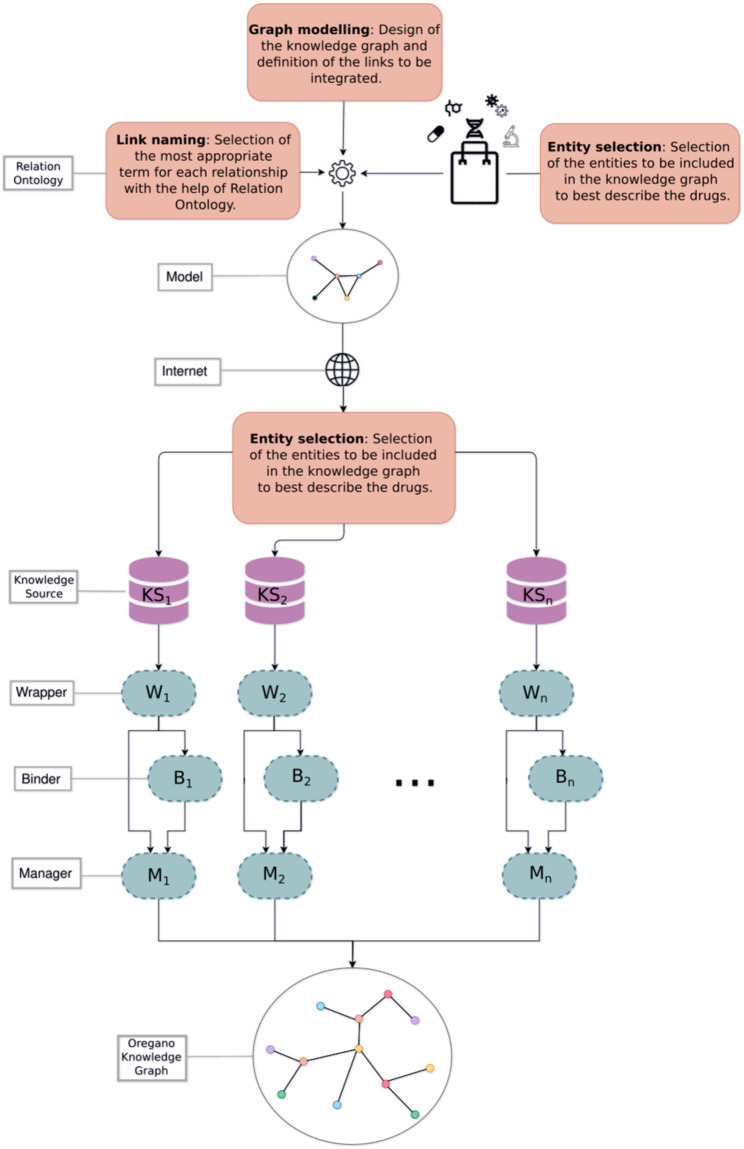
Overall workflow. After selecting the types of nodes which are of interest for drug repositioning, the labels of the links connecting these types are retrieved from the Relation Ontology^73^. The nodes and the way there are connected form the model of the knowledge graph. The sources to be integrated are chosen according to the nodes represented in the model and their free online availability. Each knowledge source has its data extracted by a dedicated wrapper. These data are processed by a binder and a manager to populate thereafter the model and thus generate the OREGANO knowledge graph.

This section describes the steps involved in building the knowledge graph (Fig. 1), i.e. the data selection, the actual construction and the data filtering performed via several stages in this work.

The information to be acquired from the knowledge sources was selected using the model that was produced and according to their ability to be integrated (subsection A.). The first version of the OREGANO knowledge graph^32^ was built using the knowledge available in Bio2RDF^12^. The utility of this resource resides in its ability to establish links and cross-references among the various knowledge bases it incorporates. Nevertheless, Bio2RDF has not been updated since 2014. Consequently, the information contained in this resource does not align with the content present in the updated iterations of the knowledge bases. In the second version of the OREGANO knowledge graph, we have thus opted to incorporate the data directly from knowledge source repositories. This approach allows us to construct a knowledge graph enriched with up-to-date information. The integration process follows the Extract, Transform and Load (ETL) principle: the wrappers extract the data (subsection B.), which are then transformed by the binders (subsection C.) and managers (subsection D.).

### A. Description of the selected knowledge sources

The process of building the OREGANO knowledge graph first required selecting the knowledge sources to be integrated. This choice was made in coherence with the previously designed OREGANO knowledge graph, which was built from data available on the Linked Open Data (LOD)^32^. The same set of sources was therefore chosen with the intention of reinforcing the integration process used to develop the first knowledge graph. The difference is that rather than retrieving the sources through the Bio2RDF proxy, we have included them in the current version as they were initially (official repository).

In this section, we introduce the four types of sources that were used in this work, i.e. those that describe: (i.) information about targets, (ii.) information about phenotypes, (iii.) information about natural compounds, and (iv.) information about drugs. In addition, we present: (v.) two additional sources, and (vi.) existing cross-references that are useful for integrating the aforementioned sources successfully.

#### i. Target-related sources

*DrugBank* is a free drug information source that was launched in 2006^38,39^. It offers a wide range of information on drugs, their targets and interactions. The knowledge about drugs is both chemical and pharmaceutical, with different labels and dosages for drugs from around the world. The use of DrugBank is open to anyone, provided they create a profile to download the data. The version used in the current work is dated back April 1, 2023. It contains respectively 16,306 drugs and 4,939 targets.

*SIDER* contains information on drugs and their side effects^40,41^. The most recent version, which was released on October 21, 2015, comprises 139,756 pairs of 1,430 drugs and 5,868 side effects. This information was derived from data from clinical trials, in which participants were observed, and any side effects were noted. Prior to these human testing phases, animal testing also contributed to the data. The resource also lists the disease indications for the included drugs.

*UniProt* is a resource about proteins. It provides in particular their sequences, annotations and information about their functions in the organism^42^. The European Bioinformatics Institute (EMBL-EBI), the SIB Swiss Institute of Bioinformatics and the Protein Information Resource (PIR) have collaborated to create and maintain it. The version we used for OREGANO was released on September 13, 2023 and aggregated information on 570,157 proteins. More specifically, UniProt contains several data sources, including UniProtKB that provides information about proteins. The latter comprises data from Swiss-Prot^43^ and TrEMBL^44^. Since the TrEMBL protein dataset is not renewed, only the Swiss-Prot data are considered in the integration process.

*Reactome* is a knowledge source containing data on biological pathways^45^. Created in 2003, it contains 2,629 human pathways binding 1,114 drugs, 14,277 proteins and 2,004 small molecules through 14,628 reactions. In this work, we used release 85, which was published in June 2023.

*PharmGKB* is a pharmacogenetic resource combining knowledge of genetic variation and drug responses^46,47^. It aims at integrating knowledge to assist clinicians and researchers in their investigations. PharmGKB has grown since it was founded in 2000 and now contains 993 drug label annotations, 201 clinical guideline annotations, 181 curated pathways, and 428 annotated drugs. The release that we integrated was made available on September 5, 2023.

#### ii. Phenotype-related source

*Human Phenotype Ontology* (HPO) is a source which is created initially in 2007 to model an ontology based on the concepts described in the Online Mendelian Inheritance in Man (OMIM)^48^. Nowadays, HPO has evolved into an ontology for diseases and the relationships between diseases and phenotypes. It contains over 13,000 terms. The HPO diseases come from the following sources: OMIM^49^, Orphanet^50^ and DECIPHER^51^. The version we have integrated is part of the June 2023 release.

#### iii. Natural compound-related source

*NPASS* is the only source not present in Bio2RDF that was included in the first version of the OREGANO knowledge graph to complete data with knowledge about natural compounds.

NPASS contains information on natural compounds and their targets in organisms^52,53^. It comprises respectively 96,481 natural compounds and 7,753 biological targets linked by 958,866 relationships. The last update was on October 4, 2023.

#### iv. Drug-related source

*Anatomical Therapeutic Chemical* (ATC) classification has been developed by the WHO Collaborating Centre. It categorises active substances according to their chemical properties and their actions in the body. Each compound is assigned a 7-character code consisting of five levels that reflect the compound’s place in the classification according to the system in which it acts and its pharmaceutical and therapeutic effects.

#### v. Additional sources

*Unified Medical Language System* (UMLS) is a resource that gathers more than 150 knowledge sources (i.e. (bio)medical coding systems and terminologies) composed of terms and relationships between them^54^. Since OMIM did not provide external links to connect it to other knowledge sources, UMLS was used to ensure proper integration of OMIM.

*Orphanet* is an online resource about rare diseases^50^. Cross-references for these diseases are not available via HPO (in which Orphanet is included), so extraction of data from Orphanet was necessary to ensure optimal HPO integration.

#### vi. Existing cross-references

**Fig. 2:**
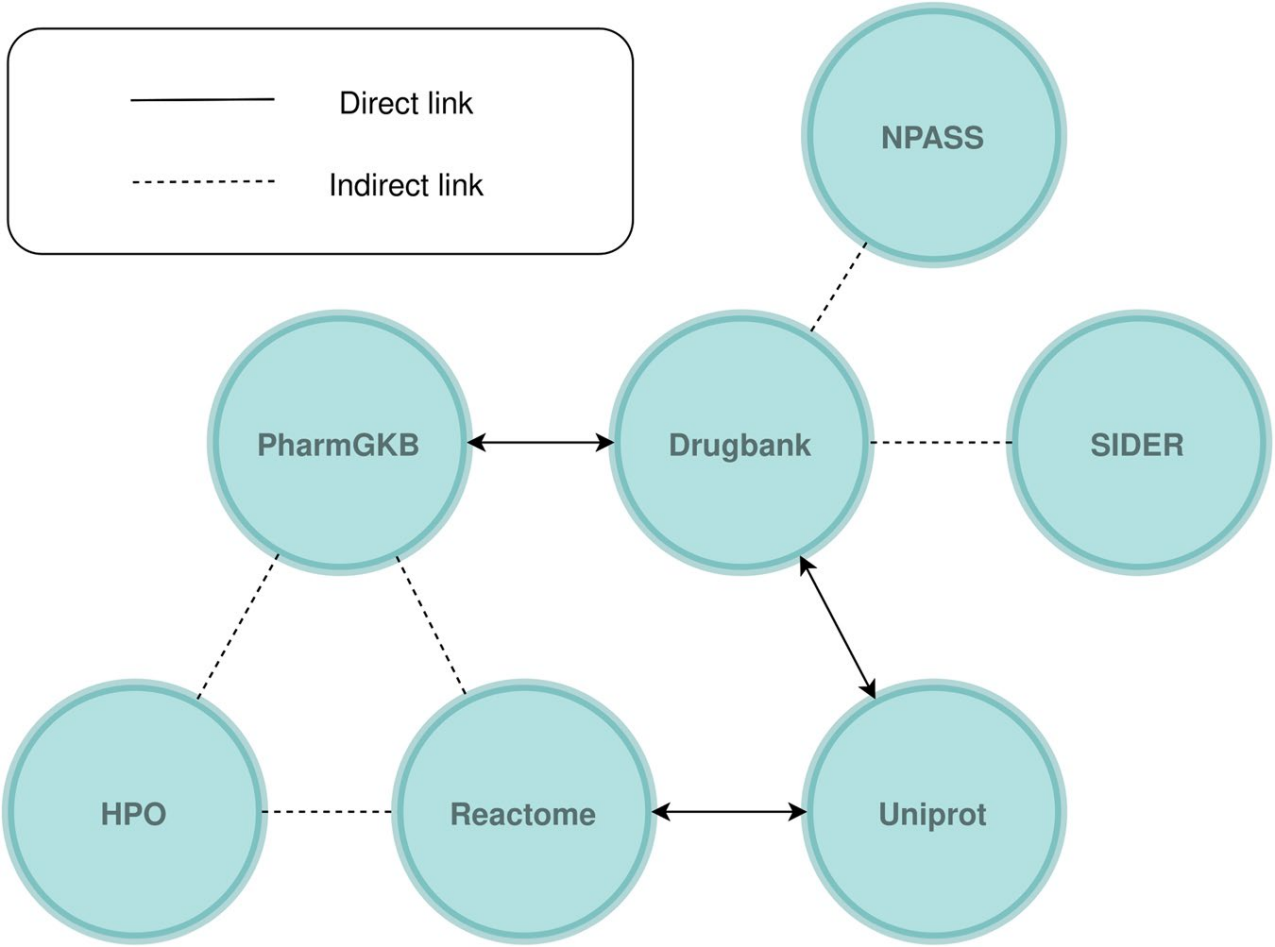
Diagram showing the different sources to be integrated in the OREGANO knowledge graph and the mappings existing between them. Solid lines denote that the source provides mappings to another source in its data, and the direction of the arrow indicates that the target source is referenced in the original source’s data. Dashed lines correspond to indirect mappings that require an intermediate source.

Finding the mappings that the knowledge sources make available is helpful to facilitate the integration process while building the knowledge graph, which involves combining the data. In Fig. 2, the different existing mappings between the seven knowledge sources are depicted. Some sources were directly connected, which made their integration simple. However, most of the information sources had indirect rather than direct mappings. Such indirect mapping entails that neither source gives cross-references to the other, but that they can still be connected by using one or more other cross-references that they both share. These mappings make it easier to find sources with related information. Since the information sources do not use the same nomenclatures, or even have the same meanings for some elements, it should be noted that establishing these cross-references requires a great deal of effort. For instance, issues can arise if duplicate proteins are found in poorly cleaned large protein libraries. In such cases, the use of an additional knowledge source as a pivot was required. As mentioned in the previous subsection, UMLS and Orphanet acted as pivots to enable the integration of OMIM and HPO, respectively.

### B. Extraction of data from knowledge sources

**Fig. 3:**
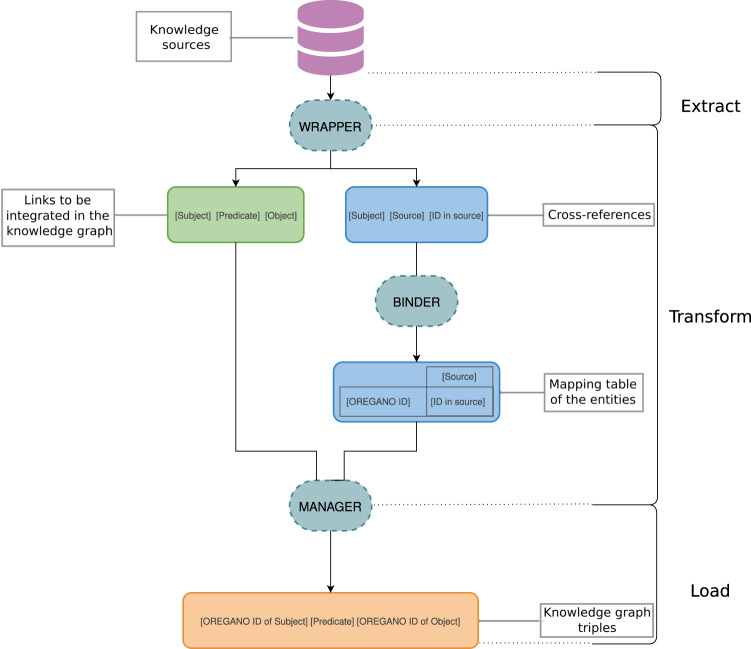
Integration workflow of a knowledge source. Data from the knowledge sources are extracted by a wrapper. The data are of two types: links to be integrated into the knowledge graph and links to external sources. The external links are used to link the data via a binder that achieves a mapping table for each type of node. It is from this mapping table that the manager converts the triples to be integrated by replacing the source codes by the OREGANO codes. This integration process follows the traditional ETL principle.

First, data were extracted from the knowledge sources that have been selected for their potential utility in drug repositioning. Then, their structure was analysed to retrieve the relevant information from each source (Table 1). The formats of the sources could differ, but each of them underwent the same overall processing. The **wrapper** (top part of Fig. 3), being the module that handled this step, first scanned the resource and then extracted the data deemed of interest for drug repositioning. Then, the resulting data were formatted into triples of the form (subject, predicate, object).

**Table 1:** Types of nodes (subject and object) and links (predicate) extracted from the different knowledge sources.

Triplets			Knowledge sources						
*Subject*	*Predicate*	*Object*	*DrugBank*	*HPO*	*NPASS*	*PharmGKB*	*Reactome*	*Sider*	*UniProt*
Compound	has_code	ATC	X						
ATC	subclass_of	ATC	X						
Compound	has_target	Target	X		X				
Compound	has_activity	Activity	X						
Compound	decreases_activity	Activity	X						
Compound	increases_activity	Activity	X						
Compound	has_effect	Effect	X						
Compound	decreases_effect	Effect	X						
Compound	increases_effect	Effect	X						
Compound	increases_efficacy	Compound	X						
Compound	decreases_efficacy	Compound	X						
Gene	causes_condition*	Disease		X		X			
Disease	has_phenotype*	Phenotype		X					
Compound	is_affecting	Gene				X			
Compound	is_substance_that_treats*	Disease				X			
Gene	acts_within*	Pathway					X		
Compound	has_indication	Indication						X	
Compound	has_side_effect	Side effect						X	
Target	gene_product_of*	Gene							X

Labels marked with an asterisk indicate the relationships selected from the Relation Ontology^67^.

The formats were quite similar, but the way the data were organised (e.g. columns for TSV or tag types and their structure for XML) and the initial data filters varied, so each knowledge source had its own wrapper.

Only information deemed pertinent to drug repositioning was included in the OREGANO knowledge graph, as the knowledge sources are overly large datasets. Since some sources provided information on the quality of their content, these were used to keep only the most relevant relations.

The NPASS data were filtered out because the targets come from a multitude of organisms (e.g. *Angelica Gigas*, *Bos taurus* or *Saccharomyces cerevisiae*). Therefore, only those relevant to humans were selected.

Only PharmGKB compounds associated with ATC codes with a length of seven digits were retained, as the other ATC codes correspond to drug classes (i.e. not to a given compound). Before integrating the links between drugs and diseases, another filtering process was applied. Indeed, the clinical annotations that highlight these links had different levels of evidence based on the associated research conducted to date. Only 1A, 1B, 2A and 2B levels were selected as they correspond to the links with the highest confidence levels (the detailed description of the different levels is available at: https://www.pharmgkb.org/page/clinAnnLevels).

For the integration of HPO, a filter was applied on the links between diseases and phenotypes. These links have a frequency of occurrence that is modelled by a calculation, as follows:

- *Excluded*: present in 0% of cases.
- *Very rare*: present in 1% to 4% of cases.
- *Occasional*: present in 5% to 29% of cases.
- *Frequent*: present in 30% to 79% of cases.
- *Very Frequent*: present in 80% to 99% of cases.
- *Obligate*: present in 100% of cases.

Below 30%, the frequency of occurrence of a symptom is thus occasional to rare. We thus made the choice to select only the phenotypes whose frequency of occurrence is greater than or equal to 30% in order to represent the diseases with the symptoms that most often describe these diseases. For the links that did not have such an indicator, the evaluation of the evidence of these links was used. The corresponding levels are: IEA (inferred from electronic annotation), PCS (published clinical study) and TAS (traceable author statement) (the detailed description of the different levels is available at: https://hpo-annotation-qc.readthedocs.io/en/latest/annotationFormat.html#phenotype-hpoa-format). Each of these levels describes how the associated link was obtained. The TAS and PCS levels have the highest confidence levels as they are derived from scientific works, while the IEA level corresponds to links inferred electronically. Therefore, only the TAS and PCS levels were kept when the frequency of occurrence was not available.

The graph integrates the HPO diseases with their OMIM codes (obtained through the UMLS). However, only the OMIM codes associated with HPO diseases were integrated to avoid overloading the knowledge graph.

UniProt is a very large knowledge source about genes and protein targets. Only the links between the nodes already present in the graph were conserved, in order to avoid introducing too many links that were not connected to the nodes of the graph.

The triples generated by the wrapper contained both edges of the knowledge graph and cross-references. The relationships were provided as input to the manager, while cross-references were directly transmitted to the binder.

### C. Fusion of nodes from distinct knowledge sources

Knowledge sources did not necessarily provide mappings to other sources. In this case, a connection had to be established between the knowledge source to be integrated and the others. The connection could be created according to external data or knowledge sources and, if possible, a new source providing this link was added/used. This was the role of the **binder**.

The binder (middle part of Fig. 3) handled the creation and merging of nodes with the addition of new identifiers specific to the OREGANO knowledge graph into a global mapping table. To do this, a cross-reference match was sought to determine whether the node was new to the data. Depending on the cross-references, multiple candidates could exist for the same node. The goal of the binder algorithm was thus to be able to choose the best match for optimal data fusion. The correspondence of nodes was evaluated based on the number of common cross-references between two nodes. In the mapping table, each column corresponds to a knowledge source and each row represents a node with an OREGANO identifier in the first column. The binder also linked any OREGANO identifier to a new node if it was not already in the graph.

### D. Constitution of the knowledge graph

The last algorithm of the integration process is performed by the **manager** algorithm. Indeed, the link manager (bottom part of Fig. 3) combined the links extracted by the wrapper and the mappings generated by the binder to format the links. Thus, the links with the original codes in the integrated knowledge sources were replaced by the OREGANO identifiers of the associated node in the mapping table.

The seven knowledge sources were integrated in a fully automatic way. The data were merged by evaluating whether the nodes were new or already present in the mapping table based on the cross-references. A node was not integrated if no linkage could be made for it.

## Data Records

**Fig. 4:**
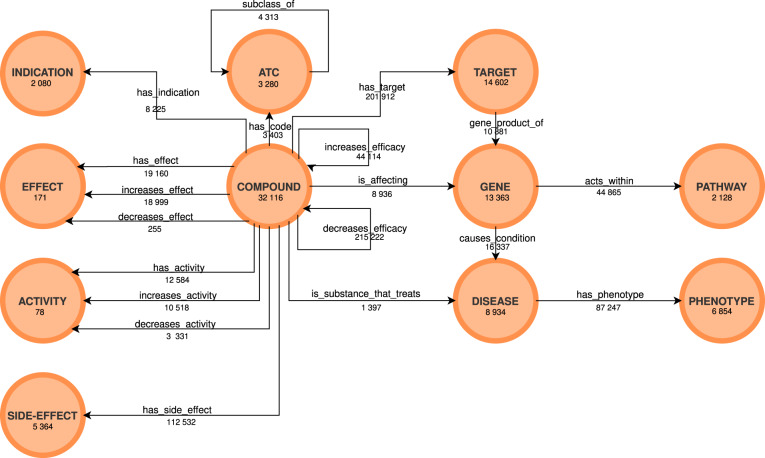
The OREGANO knowledge graph obtained after the integration of the different knowledge sources. For each node and each link, the number of occurrences in the knowledge graph is specified.

The knowledge graph (Fig. 4) is made available in TSV format in the form of three columns (subject, predicate, object). The data files are openly available on Figshare^55^ and on Zenodo^56^. The files made available are described in Table 2.

**Table 2:** Description of the files available on Figshare^55^ and Zenodo^56^.

File name	Description
OREGANO_V2.tsv	File containing all knowledge graph triples. It is composed of 3 columns: Subject; Predicate;Object
oregano_metadata_complete.ttl	The OREGANO knowledge graph in turtle format with the names and cross-references of the various integrated entities.
TARGET.tsv	Cross-reference table of the 22,096 targets.
PHENOTYPES.tsv	Cross-reference table of the 11,605 phenotypes.
DISEASES.tsv	Cross-reference table of the 18,333 diseases.
PATHWAYS.tsv	Cross-reference table of the 2,129 pathways.
GENES.tsv	Cross-reference table of the 37,794 genes.
COMPOUND.tsv	Cross-reference table of the 90,868 compounds.
INDICATIONS.tsv	Cross-reference table of the 2,714 indications.
SIDE_EFFECT.tsv	Cross-reference table of the 6,060 side-effects.
ACTIVITY.tsv	Names of the 78 activities.
EFFECT.tsv	Names of the 171 effects.

The cross-reference files are organized as follows: the column headers are the names of the sources to which the cross-references belong, and the row headers contain the name of the entity in the OREGANO graph. The first column header of each file is a key consisting of “ID_OREGANO:” followed by the number of entries in the file. The graph can be queried online through a SPARQL endpoint (http://91.121.148.199:8889/bigdata/#query).

## Technical Validation

The technical validation of the OREGANO graph was carried out in four different ways: (i) a comparison with related knowledge graphs (subsection A.), (ii) a quality assessment according to criteria defined by Chen *et al*.^57^ (subsection B.), (iii) a practical assessment with the application of an embedding algorithm exhibiting the possibility of predicting links using the knowledge graph (subsection C.), and (iv) an example of a predicted link (subsection D.).

### A. Comparison of OREGANO with other related knowledge graphs

Table 3 shows the biomedical knowledge graphs described in the Background & summary section, for which information about the number of nodes, edges, and integrated resources was available. We can see that the knowledge graphs from Zhu *et al*.^22^, PrimeKG^31^ and Hetionet^29,30^ have a significant number of nodes and relations, which is not surprising because the more knowledge sources the graph integrates and the more different types of nodes it includes, the larger the graph becomes. We can also notice that the OREGANO knowledge graph shares a similar set of data with some of these graphs, but none of them include data from PharmGKB and NPASS, and especially natural compound data. The OREGANO project thus offers a new angle to previous work by integrating this type of knowledge.

**Table 3:** Different biomedical knowledge graphs, including OREGANO, and their properties.

Knowledge graph	Type of nodes	Knowledge resources	Number of nodes	Number of links
OREGANO	Compound; Target; Gene; Disease; ATC; Phenotype; Pathway; Effect; Activity; Indication; Side-effect	DrugBank; UniProt; Human Phenotype Ontology; PharmGKB; NPASS; SIDER; Reactome	88,937	824,231
Cheng *et al*.^16^	**Drug**; **Target**	KEGG BRITE; BRENDA; SuperTarget; **DrugBank**	1,921	5,127
Zhu *et al*.^22^	Condition and Designated; **Gene**; **Protein**; **Drug**; Cell; Tissue; DATA; Chemical; **Human phenotype**; **Rare Diseases** and 32 different rare disease categories from GARD	GARD; **Orphanet**; MONDO; **OMIM**; **HPO**	3,819,623	84,223,681
Ye *et al*.^27^	**Drug**	Meyler’s Side Effects of Drugs 15th edition; Side Effects of Drugs Annuals (2007–2012); Citeline Pipeline, Thomson Reuters Partnering and GeneGo; **SIDER**	1,647	17,400
Hetionet^29,30^	Anatomy; Biological process; Cellular component; **Compound**; **Disease**; **Gene**; Molecular function; **Pathway**; Pharmacologic Class; **Side-effect**; **Symptom**	Entrez Gene; Labeledin; MEDLINE; MeSH; Pathway Interaction Database; Disease Ontology; DISEASES; DrugCentral; Gene Ontology; GWAS Catalog; **Reactome**; LINCS L1000; TISSUES; Uberon; WikiPathways; BindingDB; DisGeNET; **DrugBank**; MEDI; PREDICT; **SIDER**; Bgee; DOAF; ehrlink; Evolutionnary Rate Covariation; hetio-dag; Incomplete Interactome; Human Interactome Database; STAGEO	47,031	2,250,197
PrimeKG^31^	Biological process; **Protein**; **Disease**; **Phenotype**; Anatomy; Molecular function; **Drug**; Cellular component; **Pathway**; Exposure	The Comparative Toxicogenomics Database; DisGeNET; Disease Ontology; **DrugBank**; Drug central; Entez Gene; Gene Ontology; **Human Phenotype Ontology**; Mayo clinic; MONDO; **Orphanet**; TRANSFAC; BioGRID; STRING; MINT; IntAct; CORUM; **Reactome**; **SIDER**; Uberon; UMLS	129,375	4,050,249
OpenBioLink^74^	GO Term; **Gene**; **Disease**; **Phenotype**; Anatomy; **Drug**; **Pathway**	STRING; GO; DisGenet; **Human Phenotype Ontology**; Bgee; STITCH; CTD; DrugCentral; **SIDER**; Drug Ontology; UBERON; **UniProt**; NCBI; PubChem; **Reactome**; KEGG	184,732	9,302,547
BioKG^75^	Genetic Disorder; Disease; **Drug**; **Pathway**; **Protein**	**DrugBank**; The Human Protein Atlas; Cellosaurus; Intact; CTD; MedGen; MESH; **SIDER**; InterPro; SMPDB; **UniProt**; **Reactome**; KEGG; Hijazi20^76^	105,524	2,065,094

Types of nodes and knowledge resources common to the OREGANO knowledge graph are in bold, and those specific to OREGANO are underlined.

### B. Evaluation regarding quality assessment

In the context of knowledge graph construction, there are four aspects that are usually neglected and need to be improved, according to Abu-Salih *et al*.^58^: (i) knowledge graphs must be accessible, (ii) construction methods must be explicit and detailed, (iii) knowledge sources must be of high quality, and finally (iv) the graph must prove its efficiency and usefulness in reality. The OREGANO knowledge graph meets the first three requirements, and the fourth will be addressed in future work.

Further, to evaluate the quality of the knowledge graph, we used the framework described by Zhu *et al*.^57^. The evaluation criteria of other works mentioned in the related works^59,60^ are more suitable for knowledge graphs with a hierarchical ontological structure, which is not the case of our knowledge graph. Zhu *et al*. exposed 18 criteria for evaluating the quality of a knowledge graph. These criteria are presented in Table 4 according to four levels of consistency for each of them, ranking from perfect consistency (+++) to inconsistency (−). This table shows that for most of the criteria, the graph is of good quality. More precisely, 15 out of 18 criteria are well addressed in this current version of the OREGANO knowledge graph, while three remain to be addressed.

**Table 4:** Quality assessment table for the OREGANO knowledge graph, according to the criteria defined in Chen *et al*.^57^.

Criteria	Level of consistency	Comments
1. Triples should be concise	+++	The triples are concise, following systematically the Subject-Predicate-Object pattern. The relations between the different entities are from the Relation Ontology, so they are standardized.
2. Contextual information of entities should be captured,	+++	This item is available at several levels in the OREGANO knowledge graph. Primarily, contextual information is gathered by the relationships existing between entities. In addition to their names and cross-references, entities also possess attributes that contextualize them.
3. Knowledge graph does not contain redundant triples	+++	Since the graph does not contain any transitive relations, the triples in the OREGANO knowledge graph are not redundant.
4. Knowledge graph can be updated dynamically	++	If the formats remain identical, the data update scripts can be applied to the newly updated data of the various knowledge sources. The only remaining task is to upload these new data and initiate the integration workflow.
5. Entities should be densely connected	++	In the framework, there is no threshold for determining the quality of the density of the knowledge graph. Nevertheless, between the first and second versions, the density has been increased.
6. Relations among different types of entities should be included	+++	The knowledge graph includes different types of entities that are connected by different types of relationships.
7. Data source should be multi-field	+++	Different biomedical domains are represented by the integrated sources, including pharmacology for DrugBank and PharmGKB, genetics and proteomics for UniProt, metabolomics for Reactome, and biochemistry for NPASS and SIDER.
8. Data for constructing a knowledge graph should be in different types and from different resources	+++	The OREGANO knowledge graph brings together 7 different knowledge sources that provide different types of data on drugs, proteins, genes, diseases, etc. in different formats (TSV, XML).
9. Synonyms should be mapped and ambiguities should be eliminated to ensure reconcilable expressions	+++	In the current version of the OREGANO knowledge graph, the nodes are identified by a unique ID and they have only one plain English name (synonyms can be found by cross-referencing in the corresponding knowledge sources).
10. Knowledge graph should be organized in structured triples for easily processed by machine	+++	The knowledge graph is organised in triples and is available as is. It can also be accessible through a SPARQL endpoint.
11. The scalability with respect to the KG size	+	Increasing the size of the knowledge graph between the first and second versions did not have a significant impact on the amount of tasks performed. However, larger changes (e.g. adding new relations, new instances) could lead SPARQL query latencies and changes in the time required to provide results when the learning process or computation is performed on the graph.
12. The attributes of the entities should not be missed	++	Entities have properties that are integrated in the OREGANO knowledge graph, such as their textual names and information about their cross-references. More information could be integrated, such as drug toxicity or disease descriptions.
13. Knowledge graph should be publicly available and proprietary	+++	The knowledge graph is accessible in two locations: on GitHub in Turtle format and via a SPARQL endpoint through which users can query the OREGANO graph.
14. Knowledge graph should be authority	−	This item is complex, as our work could have authority at the local level in our research with clinicians, but at the international and national levels, our project would need to be part of larger cohorts for it to become authoritative. This is not the case at the moment, as the project is not finalised.
15. Knowledge graph should be concentrated	+++	No unnecessary information has been included in our knowledge graph; only relevant and informative information related to drug repurposing has been included.
16. The triples should not contradict with each other	+++	The knowledge graph has been analyzed by a reasoner in the Protégé tool (https://protege.stanford.edu/), and no inconsistencies were detected.
17. For domain specific tasks, the knowledge graph should be related to that field	+++	The OREGANO knowledge graph was developed with the goal of implementing link prediction techniques for drug repositioning. The graph provides data related to biochemical and pharmacological aspects, which allows this task to be performed.
18. Knowledge graph should contain the latest resources to guarantee freshness	−	The knowledge graph may or may not include the most up-to-date information from DrugBank, UniProt, Reactome, PharmGKB, and the Human Phenotype Ontology. However, as noted in criterion 4, scripts can be used to update the knowledge graph information in the future.

The level of consistency against these criteria is presented and ranked from perfect consistency (+++) to inconsistency (−). Comments specifying the level assigned are provided in the second column.

### C. Link prediction

The ultimate aim of the OREGANO project is to use its knowledge graph for drug repositioning as a knowledge base for discovering new links between molecules and targets. Node embedding algorithms can be used for link prediction over a knowledge graph^61–66^. By fitting such algorithms to a knowledge graph, they predict whether unknown edges have a high probability of existing. Each of the algorithms uses a different calculation to try to best predict the edges included in the knowledge graph (Supplementary data 1), and metrics are used to measure the probability of obtaining edges that actually exist in the graph. In this way, the knowledge graph was subjected to several node folding algorithms. To test the ability of the OREGANO knowledge graph to predict links, the whole graph was used (Supplementary data 2).

The statistical metrics used are the MRR and the Hit@N. Each one measures the possibility of obtaining a correct answer, in general for the MRR and in the first N suggestions for the Hit@N (details about these metrics are also provided in Supplementary data 1).

The PyKEEN library was used for each of the algorithms^67^. This is a Python package designed to train and evaluate knowledge graph embedding models. Each of the tested models was run for 50 epochs using the hyperparameters obtained after a parameter optimisation. Each model was run five times on five training sets from the knowledge graph. Each number is the average of the results over the five datasets.

We can see that link prediction over OREGANO performs best with the ComplEx algorithm (Table 5). The results show that with this algorithm, it is possible to obtain links with a high probability of existing at 42% of Hit@10. In other words, there is a 42% chance of obtaining a good result in the first 10 predictions.

**Table 5:** Results of the different embedding algorithms.

	TransE	TransH	TransR	RotatE	ComplEx	DistMult
MRR	0.03544	0.03988	0.13806	0.20220	0.25994	0.06254
Hit@1	0.01444	0.01616	0.07948	0.13308	0.17794	0.02528
Hit@10	0.12662	0.09002	0.23664	0.33692	0.42266	0.13606

MRR stands for Mean Reciprocal Rank and Hit@N corresponds to the probability that the correct answer will be found in the first N hits.

### D. Empirical predictions

Predictions were made on the entire knowledge graph using our best model (i.e., ComplEx as shown in Table 5). A set of link predictions was produced on the “has_target” link from the Compound entity in the knowledge graph. The top 10 predictions were retained for each compound. The results were then ranked from highest to lowest score. From the best results, we selected an example of a natural compound to illustrate the value of integrating them for drug repositioning (detailed statistics regarding the 22,676 natural compounds included in OREGANO can be found in Supplementary data 3).

One of the best predictions was “COMPOUND:10025 has_target PROTEIN:4003”. Compound 10025 is epigallocatechin-gallate (EGCg) and protein 4003 is DNA polymerase *k* (Pol *k*). EGCg is the most abundant catechin in tea. In plants, catechins are secondary metabolites with antioxidant properties, belonging to the flavonoid subgroup of polyphenols^68^. Its medicinal uses are the subject of much research; EGCg has proven anti-cancer properties, notably against lung cancer^69^. Pol *k* is a member of the Y family of DNA polymerases, which have the ability to tolerate DNA damage during replication. Thanks to its ability to copy DNA, it plays a key role in maintaining genomic integrity^70^. It is also much studied for the role it plays in certain types of cancer. In many cancers, its expression is altered, inducing a disorder of genomic stability. The identification of Pol *k* inhibitors is therefore an important area of research; Pol *k* deregulation has been demonstrated in lung cancer^71^ in particular.

EGCg has been shown to inhibit all DNA polymerases except *β*^72^. By comparing all the data available on these two molecules, a more in-depth study could be envisaged to investigate the possibility of treating certain types of cancer whose Pol *k* regulation is altered with EGCg.

### Supplementary information

Supplementary Information

## Data Availability

The code for the integration and the knowledge graph are available on the GitHub of the OREGANO project in the *Integration* folder (https://gitub.u-bordeaux.fr/erias/oregano).
