# Supplementary material for: The OREGANO knowledge graph for computational drug repurposing
Source: Sci Data. 2023 Dec 6;10:871. doi: 10.1038/s41597-023-02757-0 (PMC10700660; doi:10.1038/s41597-023-02757-0)
Supplement: Supplementary file 1 — Supplementary Information [file 41597_2023_2757_MOESM1_ESM.pdf]

Supplementary Information

Contents

Supplementary data 1: Graph embedding methods and metrics **2**

Supplementary data 2: Existing links and their origin **3**

Supplementary data 3: Detailed statistics regarding natural compounds **3**

References **4**

Supplementary data 1: Graph embedding methods and metrics

Graph embedding techniques consist in representing a graph in a space of chosen reduced dimensions. This representation allows obtaining a mathematical model of the graph and to be able to apply various oper[htbp]ations such as missing links prediction (aka graph completion).

The metrics used to evaluate predictions based on the knowledge graph are the MRR and the HIT@N.

The MRR score is comprised between zero and one; the closer the score is to one, the more likely the knowledge graph is to hit the right answer at the first suggestion. The MRR is measured as follows:

$$\frac{1}{|Q|}\sum_{i=1}^{Q}\frac{1}{rank_i}$$

with Q a sample of queries and $rank_i$ the rank of the first relevant answer of the i-th query.

The metric Hit@N is also a number between 0 and 1, which indicates the probability that the correct answer will be encountered in the first N suggestions. For a query, the Hit@N will be the fraction of relevant results in the first N results. The final Hit@N is the average of all the Hit@N for all the queries. It is often expressed as a percentage.

There are several methods for graph embedding. Among the most commonly used are TransE, TransH, TransR, Rotate, Dismult and ComplEx.

TransE method is a translational method that attempts to represent relationships by modelling them in the following way [1]:

$$e_h + e_r \approx e_t$$

with $e_h$being the head embedding, e_r the relation embedding and e_t the tail embedding. The modelling is often very efficient but it has difficulties in modelling 1-N relationships.

TransH method extends the TransE model to improve the representation of 1-N, N-1 and N-N relationships [6]. The relations are modelled by a hyperplane, the head (h) and tail (t) vectors are projected on this hyperplane. Depending on the relation, we obtain the following relation:

$$h_\perp + d_r \approx t_\perp$$

with $h_\perp$ being the projected head vector on the hyperplane, $t_\perp$ the projected tail vector on the hyperplane and d_r a vector belonging to the hyperplane.

Trans R method is an extension of TransH and treats entities and relationships as distinct objects [3], representing them in different vector spaces in the following way:

$$f\left(h,r,t\right) = -M_r e_h + e_r - M_r e_t$$

with e_h the head embedding, e_t the tail embedding, e_r the relation embedding and M_r a relation-specific projection matrix.

Rotate method models relations as rotation from head to tail in the complex space [4], in the following way:

$$f\left(h,r,t\right) = -\left\|e_h \odot e_r - e_t\right\|$$

with e_h the head embedding, e_t the tail embedding, e_r the relation embedding,

DistMult method is a simplification of RESCAL where relations are represented as diagonal matrices (W_r) [7]. The scoring function is the same as RESCAL:

$$f(h,r,t) = e_h^T W_r e_t$$

with e_h the head embedding, W_r being the diagonal relation matrices and e_t the tail embedding.

ComplEx method is an extension of the DistMult model, which models nodes and relations as vectors and the score is calculated with the Hadamard product [5] as follows:

$$f(h,r,t) = Re(e_h \odot e_r \odot e_t)$$

with $Re(x)$ the real part of the complex valued vector x, e_h the head embedding, e_r the relation embedding and e_t the tail embedding.

Supplementary data 2: Existing links and their origin

The following table shows the number and type of relationships recovered from the different knowledge sources. Labels marked with an asterisk indicate the relationships selected from the Relation Ontology [2].

**Triples**			**Knowledge sources**						
Subject	*Predicate*	*Object*	*DrugBank*	*HPO*	*NPASS*	*PharmGKB*	*Reactome*	*Sider*	*UniProt*
Compound	has_code	ATC	3,403						
ATC	subclass_of	ATC	4,313						
Compound	has_target	Target	19,253		183,286				
Compound	has_activity	Activity	12,584						
Compound	decreases_activity	Activity	3,331						
Compound	increases_activity	Activity	10,518						
Compound	has_effect	Effect	19,160						
Compound	decreases_effect	Effect	255						
Compound	increases_effect	Effect	18,999						
Compound	increases_efficacy	Compound	44,114						
Compound	decreases_efficacy	Compound	215,222						
Gene	causes_condition*	Disease		9,411		7,876			
Disease	has_phenotype*	Phenotype		88,791					
Compound	is_affecting	Gene				8,936			
Compound	is_substance_that_treats*	Disease				1,397			
Gene	acts_within*	Pathway					44,865		
Compound	has_indication	Indication						8,225	
Compound	has_side_effect	Side effect						112,532	
Target	gene_product_of*	Gene							10,881

Supplementary data 3: Detailed statistics regarding natural compounds

The following table shows detailed statistics regarding natural compounds in the OREGANO knowledge graph :

**Label of links**	**Number of links concerning the 22,676 natural compounds**
has_code	373
has_target	186,689
increases_activity	1,339
decreases_activity	444
has_activity	1,600
increases_effect	2,295
has_effect	2,315
decreases_effect	34
is_affecting	1,732
is_substance_that_treats	267
has_indication	1,062
has_side_effect	10,958

References

[1] Antoine Bordes et al. “Translating Embeddings for Modeling Multi-Relational Data”. In: *Proceedings of the 26th International Conference on Neural Information Processing Systems - Volume 2*. NIPS’13. Lake Tahoe, Nevada: Curran Associates Inc., 2013, pp. 2787–2795. DOI: https://doi.org/10.5555/2999792.2999923.

[2] Rachael P Huntley et al. “A method for increasing expressivity of Gene Ontology annotations using a compositional approach”. en. In: *BMC Bioinformatics* 15.1 (Dec. 2014), p. 155. ISSN: 1471-2105. DOI: https://doi.org/10.1186/1471-2105-15-155. URL: https://bmcbioinformatics.biomedcentral.com/articles/10.1186/1471-2105-15-155 (visited on 12/08/2021).

[3] Yankai Lin et al. “Learning Entity and Relation Embeddings for Knowledge Graph Completion”. In: *Proceedings of the AAAI Conference on Artificial Intelligence* 29.1 (Feb. 2015). DOI: https://doi.org/10.1609/aaai.v29i1.9491. URL: https://ojs.aaai.org/index.php/AAAI/article/view/9491.

[4] Zhiqing Sun et al. “RotatE: Knowledge Graph Embedding by Relational Rotation in Complex Space”. In: *arXiv* (2019). DOI: https://arxiv.org/abs/1902.10197. (Visited on 07/03/2023).

[5] Théo Trouillon et al. “Complex Embeddings for Simple Link Prediction”. In: *Proceedings of The 33rd International Conference on Machine Learning*. Ed. by Maria Florina Balcan and Kilian Q. Weinberger. Vol. 48. Proceedings of Machine Learning Research. 20–22 Jun 2016, pp. 2071–2080. DOI: https://doi.org/10.5555/3045390.3045609. URL: https://proceedings.mlr.press/v48/trouillon16.html.

[6] Zhen Wang et al. “Knowledge Graph Embedding by Translating on Hyperplanes”. In: *Proceedings of the Twenty-Eighth AAAI Conference on Artificial Intelligence*. AAAI’14. Québec City, Québec, Canada: AAAI Press, 2014, pp. 1112–1119. DOI: https://doi.org/10.5555/2893873.2894046.

[7] Bishan Yang et al. “Embedding Entities and Relations for Learning and Inference in Knowledge Bases”. In: *arXiv* (2015). DOI: http://arxiv.org/abs/1412.6575. URL: http://arxiv.org/abs/1412.6575 (visited on 06/21/2022).
